# The Effects of Intensive Neurorehabilitation on Sequence Effect in Parkinson's Disease Patients With and Without Freezing of Gait

**DOI:** 10.3389/fneur.2021.723468

**Published:** 2021-09-07

**Authors:** Alessia Putortì, Michele Corrado, Micol Avenali, Daniele Martinelli, Marta Allena, Silvano Cristina, Valentina Grillo, Luca Martinis, Stefano Tamburin, Mariano Serrao, Antonio Pisani, Cristina Tassorelli, Roberto De Icco

**Affiliations:** ^1^Movement Analysis Research Unit, IRCCS Mondino Foundation, Pavia, Italy; ^2^Department of Brain and Behavioral Sciences, University of Pavia, Pavia, Italy; ^3^Department of Public Health, Experimental Medicine and Forensic Science, University of Pavia, Pavia, Italy; ^4^Department of Neurosciences, Biomedicine and Movement Sciences, University of Verona, Verona, Italy; ^5^Department of Medico-Surgical Sciences and Biotechnologies, University of Rome Sapienza, Latina, Italy; ^6^Movement Analysis Laboratory, Policlinico Italia, Rome, Italy

**Keywords:** movement disorders, gait analysis, hypokinesia, basal ganglia, functional independence, movement analysis, parkinsonism

## Abstract

**Background:** The sequence effect (SE), defined as a reduction in amplitude of repetitive movements, is a common clinical feature of Parkinson's disease (PD) and is supposed to be a major contributor to freezing of gait (FOG). During walking, SE manifests as a step-by-step reduction in step length when approaching a turning point or gait destination, resulting in the so-called destination sequence effect (dSE). Previous studies explored the therapeutic effects of several strategies on SE, but none of them evaluated the role of an intensive rehabilitative program.

**Objectives:** Here we aim to study the effects of a 4-week rehabilitative program on dSE in patients with PD with and without FOG.

**Methods:** Forty-three patients (30 males, 70.6 ± 7.5 years old) with idiopathic PD were enrolled. The subjects were divided into two groups: patients with (PD + FOG, *n* = 23) and without FOG (PD – FOG, *n* = 20). All patients underwent a standardized 4-week intensive rehabilitation in-hospital program. At hospital admission (T0) and discharge (T1), all subjects were evaluated with an inertial gait analysis for dSE recording.

**Results:** At T0, the dSE was more negative in the PD + FOG group (−0.80 ± 0.6) when compared to the PD – FOG group (−0.39 ± 0.3) (*p* = 0.007), even when controlling for several clinical and demographic features. At T1, the dSE was reduced in the overall study population (*p* = 0.001), with a more pronounced improvement in the PD + FOG group (T0: −0.80 ± 0.6; T1: −0.23 ± 0.4) when compared to the PD – FOG group (T0: −0.39 ± 0.3; T1: −0.22 ± 0.5) (*p* = 0.012). At T1, we described in the overall study population an improvement in speed, cadence, stride duration, and stride length (*p* = 0.001 for all variables).

**Conclusions:** dSE is a core feature of PD gait dysfunction, specifically in patients with FOG. A 4-week intensive rehabilitative program improved dSE in PD patients, exerting a more notable beneficial effect in the PD + FOG group.

## Introduction

Gait impairment and freezing of gait (FOG) represent common and disabling motor features of Parkinson's disease (PD) (1). The antiparkinsonian dopaminergic therapy positively modulates a subset of gait parameters, such as speed of gait and stride length, while its impact on FOG is limited (2, 3). Growing evidence supports the efficacy of neurorehabilitation in the treatment of PD across all phases of the disease (4). Rehabilitation becomes crucial for the management of those symptoms that poorly respond to the antiparkinsonian drugs. Several strategies, such as action observation, cueing, and neuromodulation, have been applied with positive results in the rehabilitation of gait and FOG in PD, although the precise mechanisms underlying their effect are largely elusive (5–7). A deep phenotyping of gait features in individual patients as well as in different subtypes of PD will improve our knowledge on gait pathophysiology, ideally leading to tailored interventions (1). In the last years, the advent of reliable wearable devices has prompted the widespread study of several parameters of the parkinsonian gait in both clinical and research settings (1, 8–11).

The sequence effect (SE), defined as a reduction in amplitude of repetitive movements, is a common clinical feature of several tasks of PD patients both in early and advanced stages of the disease (12–14). During walking, SE manifests as a step-by-step reduction in step length when approaching a turning point or gait destination resulting in the so-called destination sequence effect (dSE) (15, 16). Experimental and clinical evidence supports the hypothesis that SE is a contributor to FOG (17, 18). Indeed, the dual requirement hypothesis suggests that FOG may be precipitated by the occurrence of SE over a constitutional reduction of stride length (gait hypokinesia) (17, 19). SE is specifically pronounced in PD patients affected by FOG, and it arises immediately before a FOG episode induced by a turning or a dual-task (16). SE may be triggered by several factors, such as an obstacle, a cognitive task, or removal of visual or integrative cues (20–22). The pathophysiology of SE is not completely elucidated. The most accepted hypothesis ascribes SE to a maladaptive basal ganglia process that fails to adjust gait pattern to environmental changes (18, 23, 24). In this view, basal ganglia are not able to provide appropriate information to the supplementary motor area, leading to a failure of internal cues and a reduction in the amplitude of automatic movement (17). Apparently, such condition is unresponsive to dopaminergic therapy supplementation, thereby representing an unmet clinical need for PD patients.

SE may be acutely modulated by several rehabilitative strategies such as split-belt treadmill (25) and reinforcement of visual cues (16), while repetitive transcranial magnetic stimulation (rTMS), antiparkinsonian dopaminergic drug administration, and attention strategies were found not to influence the SE (18, 26, 27). Kang et al. demonstrated a positive effect of exercise training on SE evaluated at upper limb in *de novo* PD patients (28), while the evidence on the role of neurorehabilitation on gait SE improvement and retention is extremely scarce (16, 28).

Here we aim to study the effects of a 4-week intensive rehabilitative gait program on the dSE in patients affected by PD with and without FOG.

## Materials and Methods

### Subjects

Forty-three patients (30 males, 70.6 ± 7.5 years old) with idiopathic PD were consecutively enrolled among those attending the Neurorehabilitation Department of the IRCCS Mondino Foundation (Pavia, Italy) between August 2019 and January 2021. Idiopathic PD was diagnosed according to the Movement Disorders Society clinical diagnostic criteria for PD (29). Inclusion criteria were as follows: age between 18 and 80 years; Hoehn and Yahr stage between I and IV; and Mini-Mental State Examination score above 24. The exclusion criteria were as follows: history of major psychiatric or other neurological conditions; comorbid rheumatological, ophthalmic, or orthopedic diseases; ongoing or previous treatment with neuroleptic drugs; and any change in dose or regimen of the antiparkinsonian therapy in the last month before enrolment. The subjects were divided into two groups: (1) patients with freezing of gait (PD + FOG) or (2) patients without freezing of gait (PD – FOG). The presence of FOG was defined by a score between 1 and 4 at either item “2.13 Freezing” or item “3.11 Freezing of gait” of the Unified Parkinson Disease Rating Scale (UPDRS) (30). In the PD + FOG group, all patients enrolled satisfied this criterion according to item “3.11 Freezing of gait” of the UPDRS.

No modifications of the drug regimen were allowed during the study.

The local ethics committee approved the study (p-20190052441), and all participants signed a written informed consent before enrollment. The trial was registered at www.clinicaltrials.gov (NCT04921748). The authors agree to share anonymized data from this analysis upon reasonable request from qualified investigators. The study was completed in February 2021.

### Study Procedures

At hospital admission (T0), the patients were evaluated by a neurologist with expertise in neurorehabilitation and movement disorders who collected a comprehensive anamnestic evaluation and performed a complete neurological examination. If the patient fulfilled inclusion/exclusion criteria and agreed to participate in the study, the baseline procedures were completed with an instrumental gait analysis and administration of a set of clinical scales that included the following: a rating of PD-related motor disability with the UPDRS part III (UPDRS-III) (30), with score of item “3.11 Freezing of gait” of the UPDRS-III scale being used as a measure of FOG; a rating of functional independence through the Functional Independence Measure (FIM) (31); and a rating of dependence in activity of daily living according to the Barthel Index (32).

After that, patients underwent a 4-week intensive rehabilitation program that consisted in individual training sessions with a physiotherapist lasting at least 90 min and delivered daily for 6 days per week in an in-hospital setting (see below for details about the strategies adopted). At the end of the rehabilitation program (T1), the patients completed the study procedure with the second instrumental gait analysis evaluation and administration of the same set of clinical scales.

### Gait Analysis and Sequence Effect Computation

The gait analysis was performed with a wearable, wireless, inertial system (BTS G-Walk, G Sensor—BTS Bioengineering S.p.A., Italy; weighs 37 g; dimensions 70 × 40 × 18 mm). The device was secured to the back of the patients between L5 and S1 vertebrae with an *ad hoc* elastic belt around the waist. The G Sensor recorded acceleration data through a triaxial accelerometer, a triaxial gyroscope, and a triaxial magnetometer (sampling rate 100 Hz). All data were transferred to a notebook and processed with a dedicated software (BTS G-Studio, BTS Bioengineering S.p.A., Italy). Gait evaluation validity and reproducibility with the G-Walk system, and inertial sensors in general, were previously demonstrated in healthy subjects as well as in PD (1, 8–11).

Gait assessment was performed by an expert technician (V.G.) in a straight hallway (35 × 2.5 m wide) without obstacles or factors that might contribute to FOG.

All subjects were recorded in the morning (between 9:00 and 11:00 a.m.) and in ON condition. After the montage of the inertial system, the subjects stood in a comfortable upright standing position, waiting to start walking. When the technician gave the order, the subjects started to walk at a comfortable pace for 20 m. The subjects independently stopped at the end of the 20-m pathway, which was clearly marked by a straight line on the ground. Gait assessments were discarded if episodes of FOG or pauses occurred during the walk. The subjects were allowed to rest according to their needs and preferences. For each subject, three optimally performed gait assessments were recorded. In the off-line analysis, the first and last strides of each walk were excluded (to avoid interference of fast acceleration/deceleration at gait starting/ending), and the remaining strides were used for the evaluation of gait parameters. For gait parameters, the average value of the three gait assessments was used. The following parameters were recorded and analyzed: speed (meters/second), cadence (steps/minute), stride length (meters), step length (meters), stride duration (seconds), single support, double support, swing duration, and stance duration (percentage stride distribution) (33).

The dSE was computed as a regression slope (β) of step length according to a previously described and validated methodology where step length is plotted against step number (16–18). The regression slope represents the decrease (negative values) or increase (positive values) in step length. In an off-line analysis, a step-by-step raw data of each gait assessment was extracted. As previously described, the last stride was excluded to avoid interference of sharp deceleration before gait ending. The length of the last six steps (*y*) ahead of the final stride was then plotted against step number (*x*), and the linear regression slope (β) was calculated as a measure of dSE (16–18, 26). In addition, the intercept (*I*) of the regression curve was used as an indirect measure of gait hypokinesia (17, 18). For each group, the relationship between gait hypokinesia and sequence effect is expressed by the function of the linear regression as follows: *y* = β (*x*) + *I*.

### Rehabilitation Treatment

All patients enrolled were treated with an in-hospital rehabilitation program focused on the rehabilitation of gait disorder in PD (7, 34). The rehabilitation program was delivered according to local regional regulation (https://www.sitilombardia.it/wp-content/uploads/2015/04/Requisiti-accreditamento-Riabilitazione_dgr_1980_14.pdf), validated rehabilitation protocols in use at our center (7, 35, 36) and in agreement with the European Physiotherapy Guideline for Parkinson Disease (file:///E:/2021/NRB%202021/eu_guideline_parkinson_guideline_for_pt_s1.pdf). The rehabilitation program was delivered by a team of physiotherapists with high expertise in neurorehabilitation and, specifically, in the rehabilitation of movement disorders. The rehabilitation treatment included passive, active-assisted, and active exercises according to evidence-based methods (37, 38). Each session consisted of isotonic and isometric exercises for the major muscles of the limbs and trunk, cardiovascular warm-up exercises, muscle stretching exercises for functional purposes, balance training exercises, specific motor exercise for hypokinesia, and 45 minutes of overground gait training. This latter was delivered without the use of devices or cueing strategies and was based on a conventional approach, which was individualized for each patient in the frame of the strategies proposed by the European Physiotherapy Guidelines for Parkinson's Disease. More specifically, we used a combination of:

- Walking taking large steps, with large amplitude arm swing;
- Walking around and over obstacles;
- Walking with sudden stops and change in direction, including backward walking;
- Walking while dual tasking;
- Walking and turning around in open or narrow spaces;
- Climbing steps or stairs.
- The rehabilitation program was similar for the PD + FOG and PD – FOG groups.

### Statistical Analysis

The sample size was calculated with freeware software G^*^Power (Version 3.1.9.6—University of Kiel, Germany) for a two-factor repeated measures ANOVA, considering as significant an effect size *f* of at least 0.30. The computation was made with the following parameters: α = 0.05; power = 95%; number of groups = 2; number of measurements = 2; correlation among repeated measures = 0.5; and non-sphericity correction = 1. The minimum sample size suggested was 40 patients, drop-outs excluded.

The Statistical Package for the Social Sciences (SPSS), version 21.0 (Windows), was used for all the computations. The Kolmogorov–Smirnov test confirmed a normal distribution of our data. Continuous variables are presented as mean ± standard deviation, while categorical data are presented as percentage (absolute number).

A univariate group comparison was performed with a Student's *t*-test for continuous variables, and statistical association among categorical variables was tested with Pearson χ^2^ test or Fisher's exact test, if appropriate. According to the results of the univariate analysis, a multivariate binary logistic regression analysis (dependent variable: PD + FOG vs. PD – FOG; covariates: clinical variables found to be significantly associated to FOG at univariate analysis) was performed according to a forward stepwise method.

A correlation analysis was performed with Pearson's test to evaluate the role of speed of gait on dSE and *I*.

Outcome measures were analyzed with a two-way repeated measures ANOVA with within-group TIME (2 levels: T0 vs. T1) and between-groups GROUP (2 levels: PD + FOG vs. PD – FOG) factors, followed by a *post-hoc* analysis with Bonferroni's correction. Gait parameters with left/right evaluations were analyzed by adding a within-group factor SIDE (left and right) to the ANOVA.

The level of significance was set at α = 0.05, always corrected for multiple comparisons where appropriate.

## Results

### Clinical and Demographic Features of Study Population

Among the 43 PD patients enrolled, 23 had freezing of gait (PD + FOG group: 19 males, 72.4 ± 5.6 years old) while the remaining 20 did not (PD – FOG group: 11 males, 68.4 ± 8.9 years old). Clinical and demographic features are presented in Table 1. When compared to PD – FOG patients, the PD + FOG group showed higher doses of levodopa equivalent daily dose (*p* = 0.020) and higher UPDRS-III score (*p* = 0.035) with lower FIM score (*p* = 0.024). These differences indicate a more advanced PD stage and higher level of disability in PD + FOG, which may be related to the longer duration of disease (*p* = 0.025).

**Table 1:** Baseline clinical and demographic features of the study population.

		**All patients**	**PD + FOG**	**PD – FOG**	***p*-value**
*N*		43	23	20	–
Age (years)		70.5 ± 7.5	72.4 ± 5.6	68.4 ± 8.9	0.080
Sex (male)			82.6% (19)	55.0% (11)	0.094
PD duration (years)		9.6 ± 6.2	11.5 ± 6.1	7.3 ± 5.6	**0.025**
Most affected side at PD onset	Left	62.8% (27)	52.2% (12)	75.0% (15)	0.206
	Right	37.2% (16)	47.8% (11)	25.0% (5)	
Type of PD at onset	Akinetic-rigid	48.8% (21)	39.1% (9)	60.0% (12)	0.227
	Tremor-dominant	51.2% (22)	60.9% (14)	40.0% (8)	
Patients with festination		23.3% (10)	30.4% (7)	15.0% (3)	0.294
Patients with frequent falls		34.9% (15)	39.1% (9)	30.0% (6)	0.749
Ongoing antiparkinsonian therapy	Levodopa	95.3% (41)	95.7% (22)	95.0% (19)	0.428
	Dopamine agonist	69.8% (30)	78.3% (18)	60.0% (12)	
	COMT inhibitor	2.3% (1)	4.3% (1)	0.0% (0)	
	MAO-B inhibitor	23.2% (10)	30.4% (7)	15% (3)	
Hoehn & Yahr stage	Stage I	25.6% (11)	17.4% (4)	35.0% (7)	0.141
	Stage II	37.2% (16)	30.4% (7)	45.0% (9)	
	Stage III	32.6% (14)	43.5% (10)	20.0% (4)	
	Stage IV	4.7% (2)	8.7% (2)	0.0% (0)	
Levodopa equivalent daily dose (mg)		859.4 ± 306.6	959.1 ± 301.2	744.8 ± 277.3	**0.020**
UPDRS-III total score		31.1 ± 9.9	34.1 ± 10.3	27.7 ± 8.5	**0.035**
FIM score		91.7 ± 12.1	87.8 ± 13.1	96.1 ± 9.4	**0.024**
Barthel Index		68.7 ± 14.4	65.0 ± 15.5	73.0 ± 11.9	0.069

*COMT, catechol-O-methyltransferase; MAO-B, monoamine oxidase B; PD + FOG, patients affected by freezing of gait; PD – FOG, patients not affected by freezing of gait; PD, Parkinson's disease; FOG, freezing of gait; UPDRS-III, Unified Parkinson's Disease Rating Scale part III (motor examination); FIM, Functional Independence Measure. Table entries in bold indicate significant comparisons. Patients with frequent falls were defined as those who experienced at least five falls in the past 6 months (39)*.

### Sequence Effect and Gait Analysis Parameters at Baseline

The dSE was more negative (namely, the progressive step length reduction was markedly reduced when approaching destination) in the PD + FOG group (−0.80 ± 0.6) when compared to the PD – FOG group (−0.39 ± 0.3) (*p* = 0.007). By contrast, the intercept *I* was comparable between study groups (PD + FOG: 44.7 ± 11.3; PD – FOG: 50.5 ± 14.4; *p* = 0.146).

At baseline, the PD + FOG and PD – FOG groups were comparable in speed, cadence, stride duration, and percentage distribution of stance, swing, double support, and single support. By contrast, left and right stride lengths were shorter in the PD + FOG group when compared to patients without FOG (*p* = 0.046 for both left and right strides) (Table 2).

**Table 2:** Baseline gait features of the study population.

	**All patients**	**PD + FOG**	**PD – FOG**	***p*-value**
Sequence effect (dSE)	−0.61 ± 0.5	−0.80 ± 0.6	−0.39 ± 0.3	**0.007**
Intercept (*I*)	47.4 ± 13.0	44.7 ± 11.3	50.5 ± 14.4	0.146
Speed (m/s)	0.73 ± 0.28	0.66 ± 0.25	0.81 ± 0.32	0.107
Cadence (steps/min)	101.59 ± 16.71	102.63 ± 19.24	100.56 ± 14.19	0.694
*Left side*				
Stride duration (s)	1.23 ± 0.21	1.23 ± 0.24	1.23 ± 0.18	0.981
Stride length (m)	0.87 ± 0.29	0.79 ± 0.22	0.97 ± 0.34	**0.046**
Stance (%)	60.46 ± 3.85	61.26 ± 3.66	59.54 ± 3.94	0.146
Swing (%)	39.53 ± 3.85	38.73 ± 3.66	40.45 ± 3.94	0.146
Double support (%)	10.26 ± 2.52	10.41 ± 2.65	10.08 ± 2.43	0.676
Single support (%)	39.21 ± 3.67	39.38 ± 3.54	39.01 ± 3.90	0.746
*Right side*				
Stride duration (s)	1.24 ± 0.21	1.23 ± 0.24	1.24 ± 0.18	0.966
Stride length (m)	0.87 ± 0.29	0.79 ± 0.22	0.97 ± 0.34	**0.046**
Stance (%)	61.78 ± 6.65	60.66 ± 3.61	63.07 ± 8.91	0.241
Swing (%)	39.02 ± 3.88	39.17 ± 3.79	38.85 ± 4.07	0.789
Double support (%)	10.96 ± 2.81	11.48 ± 2.88	10.36 ± 2.68	0.199
Single support (%)	39.61 ± 3.94	38.75 ± 3.67	40.61 ± 4.09	0.123

*PD + FOG, patients affected by freezing of gait; PD – FOG, patients not affected by freezing of gait; PD, Parkinson's disease. Intercept (I) is the measure of gait hypokinesia. Table entries in bold indicate significant comparisons*.

A multivariate binary logistic regression analysis was performed to evaluate the role of clinical and gait features (covariates: PD duration, UPDRS-III score, FIM score, levodopa equivalent dose, dSE, and left and right stride length) that were found to be significantly associated to FOG (dependent variable: PD + FOG group vs. PD – FOG group) in univariate analysis. The only variables that survived the multivariate regression (*R*^2^: 0.483) were dSE (*p* = 0.007; *B* = 3.795) and levodopa equivalent daily dose (*p* = 0.006; *B* = −0.005).

### Effect of the Rehabilitation Program on Sequence Effect and Gait Hypokinesia

**Figure 1:**
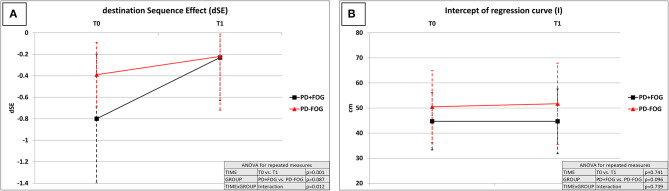
Effect of the rehabilitation program on destination sequence effect (dSE) and hypokinesia (measured by intercept “*I*”). dSE, destination sequence effect; *I*, intercept of regression curve (measure of gait hypokinesia); T0, hospital admission; T1, end of the 4-week rehabilitation program—hospital discharge; PD + FOG, patients with Parkinson's disease with freezing of gait (black lines—*n* = 23); PD – FOG, patients with Parkinson's disease without freezing of gait (red lines—*n* = 20). Two-way repeated measures ANOVA with within-group TIME (T0 vs. T1) and between-groups GROUP (PD + FOG vs. PD – FOG) factors. **(A)** dSE: TIME: *p* = 0.001; GROUP: *p* = 0.087; TIME × GROUP: *p* = 0.012. The dSE improved at the end of the rehabilitation period in the overall study population, with a more pronounced improvement in the PD + FOG group when compared to the PD–FOG group. At T1, the dSE was comparable between the PD + FOG and PD – FOG groups (*p* = 0.789). **(B)** *I*: TIME: *p* = 0.741; GROUP: *p* = 0.096; TIME × GROUP: *p* = 0.739. Gait hypokinesia measured by *I* was not significantly modified by the rehabilitative intervention, regardless of the presence of FOG.

The dSE improved at the end of the rehabilitation period in the overall study population (T0: −0.63 ± 0.5; T1: −0.23 ± 0.4; factor TIME: *p* = 0.001). A significant TIME × GROUP interaction (*p* = 0.012) was consistent with a more pronounced improvement of dSE in the PD + FOG group (T0: −0.80 ± 0.6; T1: −0.23 ± 0.4; *post-hoc* T0 vs. T1: *p* = 0.001) when compared to the PD – FOG group (T0: −0.39 ± 0.3; T1: −0.22 ± 0.5; *post-hoc* T0 vs. T1: *p* = 0.173). At T1, the dSE was comparable between the PD + FOG and PD – FOG groups (factor GROUP: *p* = 0.087; *post-hoc* PD + FOG vs. PD – FOG at T1: *p* = 0.789) (Table 3; Figure 1).

**Table 3:** Effect of the rehabilitation program on gait parameters and clinical scale scores.

	**T0**		**T1**		**ANOVA for repeated measures**		
	**PD + FOG**	**PD – FOG**	**PD + FOG**	**PD – FOG**	**TIME**	**GROUP**	**TIME × GROUP**
*Gait analysis parameters*							
Sequence effect (dSE)	−0.80 ± 0.6	−0.39 ± 0.3	−0.23 ± 0.4	−0.22 ± 0.4	**0.001**	0.087	**0.012**
Intercept (*I*)	44.7 ± 11.3	50.5 ± 14.4	44.7 ± 12.9	51.7 ± 16.2	0.741	0.096	0.739
Speed (m/s)	0.66 ± 0.25	0.81 ± 0.32	0.87 ± 0.29	0.95 ± 0.30	**0.001**	0.170	0.412
Cadence (steps/min)	102.63 ± 19.24	100.56 ± 14.19	107.38 ± 18.86	106.29 ± 14.85	**0.001**	0.757	0.714
Stride duration (s)	1.23 ± 0.24	1.23 ± 0.18	1.16 ± 0.22	1.16 ± 0.17	**0.001**	0.977	0.885
Stride length (m)	0.79 ± 0.22	0.97 ± 0.34	0.97 ± 0.31	1.06 ± 0.29	**0.004**	0.089	0.333
Stance (%)	60.96 ± 3.63	61.30 ± 6.42	61.31 ± 3.85	61.34 ± 5.75	0.797	0.803	0.841
Swing (%)	38.95 ± 3.72	39.65 ± 4.00	38.82 ± 3.39	39.56 ± 3.50	0.744	0.224	0.943
Double support (%)	10.94 ± 2.76	10.22 ± 2.55	11.09 ± 2.60	10.59 ± 2.49	0.406	0.278	0.721
Single support (%)	39.06 ± 3.60	39.81 ± 4.49	36.56 ± 4.67	39.46 ± 3.16	0.850	0.620	0.278
*Clinical scale scores*
UPDRS-III total score	34.1 ± 10.3	27.7 ± 8.5	26.0 ± 9.0	22.4 ± 6.5	**0.001**	0.058	0.130
FIM score	87.8 ± 13.1	96.1 ± 9.4	101.8 ± 12.8	108.3 ± 9.0	**0.001**	**0.031**	0.436
Barthel Index	65.0 ± 15.5	73.0 ± 11.9	83.0 ± 14.3	89.7 ± 10.1	**0.001**	**0.044**	0.747

*PD + FOG, patients affected by freezing of gait; PD – FOG, patients not affected by freezing of gait; PD, Parkinson's disease; FOG, freezing of gait. UPDRS-III, Unified Parkinson's Disease Rating Scale part III (motor examination); FIM, Functional Independence Measure. Intercept (I) is the measure of gait hypokinesia. Table entries in bold indicate significant comparisons*.

By contrast, intercept *I*, a measure of gait hypokinesia, was not significantly modified by the rehabilitative intervention (factor TIME: *p* = 0.741), regardless of the presence of FOG (factor GROUP: *p* = 0.096; TIME × GROUP interaction: *p* = 0.739) (Table 3; Figure 1). Speed of gait did not correlate with dSE at T0 (*p* = 0.268) as well as at T1 (*p* = 0.663). By contrast, speed of gait was significantly associated with intercept *I* at T0 (Pearson: 0.831, *p* = 0.001) and at T1 (Pearson: 0.834, *p* = 0.001).

**Figure 2:**
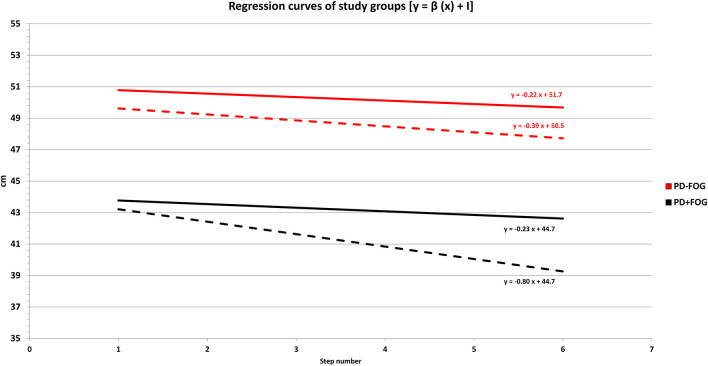
Regression curves of study groups before (T0) and after (T1) the 4-week rehabilitation program. PD + FOG: patients with Parkinson's disease with freezing of gait (black lines—*n* = 23). PD – FOG: patients with Parkinson's disease without freezing of gait (red lines—*n* = 20). The length of the last six steps (*y*-axis) ahead of the final stride of gait was plotted against step number (*x*-axis). The linear regression slope (β) was calculated as a measure of the destination sequence effect (dSE). The intercept (*I*) of the regression curve represented an indirect measure of gait hypokinesia. For each group, the relationship between gait hypokinesia and sequence effect is expressed by the function of the linear regression as follows: *y* = β (*x*) + *I*. Dashed lines: regression curves of study groups at T0 (hospital admission). Continuous lines: regression curves of study groups at T1 (end of the 4-week rehabilitation program—hospital discharge). Descriptively, at T0 (hospital admission) the PD + FOG group (black dashed line) was characterized by a greater negative slope (more pronounced dSE) and a lower position (reduced step length and pronounced gait hypokinesia) when compared to the PD – FOG group (red dashed line). At the end of the 4-week rehabilitation program (T1), we found a reduction of the linear regression slope (β), which is consistent with a dSE improvement in the overall study population. This improvement was more pronounced in the PD + FOG group (black continuous line), leading to a slope of the regression curve almost parallel to the PD – FOG group (red continuous line).

The average regression curves of the two study groups are presented in Figure 2.

### Effect of Rehabilitation Program on Other Gait Analysis Parameters

When the overall study population was considered, at the end of the rehabilitation program (T1), we described a significant improvement in speed (factor TIME: *p* = 0.001), cadence (factor TIME: *p* = 0.001), stride duration (factor TIME: *p* = 0.001), and stride length (factor TIME: *p* = 0.004). The modification of these parameters was not associated to the presence/absence of FOG (factor GROUP and interaction TIME × GROUP: *p* > 0.050 for all comparisons).

Stance, swing, double support, and single support of gait were not modified by the rehabilitative intervention (factor TIME: *p* > 0.050 for all comparisons) (Table 3). For all gait parameters with left/right evaluations, factor SIDE was not significant, and it was not considered for results interpretation.

### Effect of Rehabilitation Program on Clinical Scale Scores

When the overall study population was considered, at the end of the rehabilitation program (T1), we documented a significant improvement in UPDRS-III (factor TIME: *p* = 0.001), FIM (factor TIME: *p* = 0.001), and Barthel Index scores (factor TIME: *p* = 0.001) (Table 3).

The modification of FIM, UPDRS-III, and Barthel Index scores were not associated to the presence/absence of FOG (interaction TIME × GROUP: *p* > 0.050 for all comparisons) (Table 3).

In the PD + FOG group, the score of item “3.11 Freezing of gait” of the UPDRS-III scale improved at T1 (T0: 1.4 ± 1.1; T1: 0.6 ± 0.7; *p* = 0.001).

## Discussion

In the present study, we evaluated the role of a 4-week intensive rehabilitative program with overground gait training on the dSE in PD patients with and without FOG. The dSE is a physiological step-by-step reduction in step length that occurs when a subject approaches the end of gait, probably representing a safety strategy toward a planned gait termination (15). dSE may become pathological when particularly pronounced as in PD and specifically in patients with FOG (16–18).

The main results of our study may be summarized as follows: (i) dSE is more pronounced in PD patients with FOG (PD + FOG group), being the association with FOG confirmed by a multivariate analysis controlled for several variables; (ii) the rehabilitative program positively modulated dSE in PD patients, with a beneficial effect more pronounced in the PD + FOG group that achieved dSE values comparable to the PD – FOG group at the end of rehabilitation; and (iii) neurorehabilitation plays a pivotal role in the management of some gait disorders in PD, as demonstrated by the improvement in speed, cadence, stride duration, and stride length at T1 in the overall PD cohort.

The baseline clinical and demographic parameters of our study population are in line with available literature (16, 17, 21). Indeed, PD + FOG patients had longer disease duration, more severe motor disability (UPDRS-III score), lower functional independence (FIM score), higher levodopa equivalent daily dose, and shorter stride length. The percentage of patients with frequent falls or festination was similar between groups, although these associated clinical features were numerically more prevalent in the PD + FOG group, as expected (18, 39, 40).

The association between SE and a FOG phenotype is not a novel finding. Chee et al. demonstrated greater SE in PD + FOG patients when compared to the PD – FOG group and healthy controls (17). In line with our results, Cao et al. reported greater SE in PD + FOG patients when compared to PD – FOG patients, even when the analysis was controlled for clinical features (e.g., disease duration, UPDRS-III, and Hoehn and Yahr stage) and gait (step length and step length variability) (16).

Previous studies explored the therapeutic effects of several strategies on SE, but none of them evaluated the role of an intensive rehabilitative program (16, 25, 28). Cao et al. confirmed that visual cueing delivered through floor transverse strips could effectively reduce FOG episodes by improving step length and dSE, leading to comparable dSE values between PD + FOG and PD – FOG patients under acute cueing conditioning (16). According to their hypothesis, only strategies that eliminate SE may treat FOG successfully. This idea was supported by the fact that wearable laser lights, although improving step length, did not improve dSE and failed to reduce FOG episodes. This hypothesis fits well with the dual requirement theory that suggests that FOG may be precipitated by the occurrence of SE over a constitutional reduction of stride length (gait hypokinesia) (17, 19).

Fasano et al. studied a cohort of PD patients during split-belt walking training in three conditions: (i) with the two belts moving at the same speed (tied configuration); (ii) with reduced speed of the belt on the side of the lower limb with shorter step length; and (iii) with reduced speed of the belt on the side of the lower limb with longer step length (25). They demonstrated a worsening in SE in the late phase of the condition where the belt speed of the best side (longer step length) was slowed (25). This observation supports the idea that gait asymmetry may be a precipitating factor for SE and FOG, probably as a result of a shared maladaptive motor behavior that results in a defective scaling of movement amplitude (25, 41).

Another interesting observation was provided by Chee et al. (17). Experimentally reducing the step length of PD patients up to 75% of the normalized baseline step length led to a worsening of SE only in PD + FOG patients, but not in PD – FOG and healthy controls groups (17). This is in line with the more pronounced improvement of dSE we observed in PD + FOG patients at the end of the neurorehabilitation program. These findings suggest that SE not only appears to be a salient feature of PD + FOG patients but also seems more amenable to modulation by environmental interventions in this phenotype when compared to PD – FOG patients and healthy controls. The added value of our study is the demonstration of gait improvement to a rehabilitation protocol that can be applied in the clinical setting, instead of an experimental condition.

SE is poorly affected by levodopa administration (27, 42). Indeed, PD patients in ON medication state showed higher SE than healthy controls, with this impairment being reversed by visual feedback administration (27). This observation was in line with the results by Iansek et al. (18). In PD patients with FOG and festination, SE was alleviated by visual cues administration, but not by dopaminergic medication or pure attentional strategies (18). Kang et al. demonstrated that motor training improved bradykinesia and the SE at the upper limb in patients with *de novo* PD in OFF state. Conversely, the SE did not improve with exercise when tested under dopaminergic medication (28). rTMS delivered over the supplementary motor area (SMA) improved several gait parameters in PD (namely, speed, cadence, and step count) and exerted a long-lasting beneficial effect on FOG without directly influencing the SE (26).

The pathophysiology underlying the SE is yet to be completely elucidated. A major role seems to be played by the inability of the basal ganglia/SMA circuitry to provide a proper internal timing and cue production as well as an adequate movement scaling (12, 16, 27, 43, 44). Although isolated motor tasks may be preserved in PD, the typical internal cueing deficit may impact the automatic execution of a repetitive motor plan, leading to a gradual amplitude reduction and SE (12, 45).

If the basal ganglia/SMA system appears to be crucial in the SE generation, it is difficult to hypothesize that our rehabilitative interventions might exert a positive modulation of SE trough this pathway. This idea is corroborated by the lack of efficacy on SE of either L-dopa administration, which mainly acts at the basal ganglia level, or rTMS direct modulation of SMA (18, 26–28, 42).

By contrast, the consistent effects of the cueing and rehabilitative strategies suggest the SE may be modulated trough activation of the so-called lateral system, which includes the parietal and premotor cortices, and the cerebellum (2, 46, 47). Indeed, activation of the lateral system, and specifically of the cerebellum, by means of exercise or external environmental pacing may induce motor improvement by compensating the hypoactive internal rhythmic signal generator in PD (43, 44, 48).

Technical and methodological issues (namely, a different gait analysis tool and the evaluation of SE in different gait phases) may explain the observed discrepancies. Another concern may exist on the possible association between the modifications of dSE and speed of gait improvement at the end of rehabilitation; indeed, several gait parameters appear to be speed dependent (49). In our study, the following observations contradict this hypothesis: (i) a correlation analysis excluded a direct association between dSE and speed of gait at T0 as well as T1; (ii) speed of gait was not significantly different between the PD + FOG and PD – FOG groups; and (iii) speed of gait improvement was comparable between PD + FOG and PD – FOG patients, while dSE was specifically modified in the PD + FOG group.

The main strength of this study is that it documents dSE improvement with rehabilitation and opens some new avenues for rehabilitation applications to patients with FOG and SE.

Our report has several limitations that must be acknowledged. While we extensively recorded anamnestic and clinical data of our PD patients, we did not rate the severity of FOG with a dedicated clinical scale such as the freezing of gait questionnaire. Our primary aim was to evaluate the role of a rehabilitation program on the dSE and not on FOG, but, based on present results, we believe that a more precise phenotyping of FOG may be relevant in future studies. Indeed, with this limitation, it is difficult to clearly state the real relationship between dSE improvement and FOG. In the present study, we enrolled a population without cognitive impairment (MMSE > 24), which of course limits generalization to a PD population with different clinical features. In addition, the absence of a control group and a comprehensive rehabilitation program does not allow to explore whether dSE improvement can be ascribed to intensity of rehabilitation. Finally, we demonstrated a role of neurorehabilitation on gait dSE at the end of a 4-week rehabilitation program, but we did not assess the long-term effects, the duration of dSE improvement after neurorehabilitation, and whether our findings result in reduced risk of falls, which are frequent complications of FOG.

## Conclusions

Gait dSE represents a pathogenetic feature of PD, being more pronounced in patients with FOG. Our results demonstrate that a 4-week intensive, in-hospital, rehabilitation program significantly reduces dSE in PD patients with FOG. dSE improvement may reduce FOG episodes by targeting one of the underlying pathophysiological mechanisms discussed above. Further research is needed to better address the relationship between SE and FOG as well as the long-term effects of neurorehabilitation on these parameters.

## Data Availability Statement

The raw data supporting the conclusions of this article will be made available by the authors, upon reasonable request.

## Ethics Statement

The studies involving human participants were reviewed and approved by local ethics committee of Pavia (code: p-20190052441). The patients/participants provided their written informed consent to participate in this study.

## Author Contributions

APu: study concept and design, patients enrolment, and writing of the first draft. MC: acquisition of data, patients enrolment, and drafting/revision of the manuscript for content. MAv, DM, MAl, and SC: patients enrolment and drafting/revision of the manuscript for content. VG: major role in acquisition of data and off-line data analysis. LM: major role in patients rehabilitation treatment and drafting/revision of the manuscript for content. ST and MS: study concept and design, interpretation of data, and drafting/revision of the manuscript for content. APi: interpretation of data and drafting/revision of the manuscript for content. CT: study concept and design, patients enrolment, interpretation of data, and drafting/revision of the manuscript for content. RDI: study concept and design, acquisition of data, statistical analysis and interpretation of data, and writing of the first draft. All authors contributed to the article and approved the submitted version.

## Funding

This study was funded by the Italian Ministry of Health (Ricerca Corrente 2018–2020).

## Conflict of Interest

APi holds grants that are not related to the subject of the present study, and he reports no biomedical financial interests or potential conflicts of interest. CT holds grants and received scientific consulting fees from drug companies that are not related to the subject of the present study. The remaining authors declare that the research was conducted in the absence of any commercial or financial relationships that could be construed as a potential conflict of interest. The reviewer AS declared a shared affiliation, with no collaboration, with one of the authors MS to the handling Editor.

## Publisher's Note

All claims expressed in this article are solely those of the authors and do not necessarily represent those of their affiliated organizations, or those of the publisher, the editors and the reviewers. Any product that may be evaluated in this article, or claim that may be made by its manufacturer, is not guaranteed or endorsed by the publisher.

## References

[1] Mirelman A Bonato P Camicioli R Ellis TD Giladi N Hamilton JL. Gait impairments in Parkinson's disease. Lancet Neurol. (2019) 18:697–708. 10.1016/S1474-4422(19)30044-4 30975519

[2] Peterson DS Horak FB. Neural control of walking in people with Parkinsonism. Physiology. (2016) 31:95–107. 10.1152/physiol.00034.2015 26889015 PMC4888974

[3] Schaafsma JD Balash Y Gurevich T Bartels AL Hausdorff JM Giladi N. Characterization of freezing of gait subtypes and the response of each to levodopa in Parkinson's disease. Eur J Neurol. (2003) 10:391–8. 10.1046/j.1468-1331.2003.00611.x 12823491

[4] Martignon C Pedrinolla A Ruzzante F Giuriato G Laginestra FG Bouça-Machado R. Guidelines on exercise testing and prescription for patients at different stages of Parkinson's disease. Aging Clin Exp Res. (2021) 33:221–46. 10.1007/s40520-020-01612-1 32514871

[5] Pelosin E Avanzino L Bove M Stramesi P Nieuwboer A Abbruzzese G. Action observation improves freezing of gait in patients with Parkinson's disease. Neurorehabil Neural Repair. (2010) 24:746–52. 10.1177/1545968310368685 20453155

[6] Nardone R Versace V Brigo F Golaszewski S Carnicelli L Saltuari L. Transcranial magnetic stimulation and gait disturbances in Parkinson's disease: a systematic review. Neurophysiol Clin. (2020) 50:213–25. 10.1016/j.neucli.2020.05.002 32620273

[7] De Icco R Tassorelli C Berra E Bolla M Pacchetti C Sandrini G. Acute and chronic effect of acoustic and visual cues on gait training in Parkinson's Disease: a randomized, controlled study. Parkinsons Dis. (2015) 2015:1–9. 10.1155/2015/978590 26693384 PMC4674608

[8] Zago M Sforza C Pacifici I Cimolin V Camerota F Celletti C. Gait evaluation using inertial measurement units in subjects with Parkinson's disease. J Electromyogr Kinesiol. (2018) 42:44–8. 10.1016/j.jelekin.2018.06.009 29940494

[9] Vítečková S Horáková H Poláková K Krupička R RuŽička E BroŽová H. Agreement between the GAITRite R System and the Wearable Sensor BTS G-Walk R for measurement of gait parameters in healthy adults and Parkinson's disease patients. PeerJ. (2020) 22:e8835. 10.7717/peerj.8835 32509441 PMC7247524

[10] Schwesig R Leuchte S Fischer D Ullmann R Kluttig A. Inertial sensor based reference gait data for healthy subjects. Gait Posture. (2011) 33:673–8. 10.1016/j.gaitpost.2011.02.023 21458270

[11] Pau M Leban B Collu G Migliaccio GM. Effect of light and vigorous physical activity on balance and gait of older adults. Arch Gerontol Geriatr. (2014) 59:568–73. 10.1016/j.archger.2014.07.008 25127848

[12] Kang SY Wasaka T Shamim EA Auh S Ueki Y Lopez GJ. Characteristics of the sequence effect in Parkinson's disease. Mov Disord. (2010) 25:2148–55. 10.1002/mds.23251 20669182 PMC4782591

[13] Benecke R Rothwell JC Dick JPR Day BL Marsden CD. Disturbance of sequential movements in patients with parkinson's disease. Brain. (1987) 110:361–79. 10.1093/brain/110.2.361 3567527

[14] Kang SY Wasaka T Shamim EA Auh S Ueki Y Dang N. The sequence effect in de novo Parkinson's disease. J Mov Disord. (2011) 4:38–40. 10.14802/jmd.11006 24868390 PMC4027704

[15] Virmani T Pillai L Glover A Doerhoff SM Williams DK Garcia-Rill E. Impaired step-length setting prior to turning in Parkinson's disease patients with freezing of gait HHS Public Access. Mov Disord. (2018) 33:1823–5. 10.1002/mds.27499 30306629 PMC6358011

[16] Cao SS Yuan XZ Wang SH Taximaimaiti R Wang XP. Transverse strips instead of wearable laser lights alleviate the sequence effect toward a destination in parkinson's disease patients with freezing of gait. Front Neurol. (2020) 11:838. 10.3389/fneur.2020.00838 32903360 PMC7434927

[17] Chee R Murphy A Danoudis M Georgiou-Karistianis N Iansek R. Gait freezing in Parkinson's disease and the stride length sequence effect interaction. Brain. (2009) 132:2151–60. 10.1093/brain/awp053 19433440

[18] Iansek R Huxham F McGinley J. The sequence effect and gait festination in parkinson disease: contributors to freezing of gait? Mov Disord. (2006) 21:1419–24. 10.1002/mds.20998 16773644

[19] Iansek R Danoudis M. Freezing of gait in Parkinson's disease: its pathophysiology and pragmatic approaches to management. Mov Disord Clin Pract. (2017) 4:290–7. 10.1002/mdc3.12463 30868095 PMC6407046

[20] Rahman S Griffin HJ Quinn NP Jahanshahi M. The factors that induce or overcome freezing of gait in Parkinson's disease. Behav Neurol. (2008) 19:127–36. 10.1155/2008/456298 18641432 PMC5452481

[21] Nieuwboer A Dom R De Weerdt W Desloovere K Fieuws S Broens-Kaucsik E. Abnormalities of the spatiotemporal characteristics of Gait at the onset of freezing in Parkinson's disease. Mov Disord. (2001) 16:1066–75. 10.1002/mds.1206 11748737

[22] Spildooren J Vercruysse S Desloovere K Vandenberghe W Kerckhofs E Nieuwboer A. Freezing of gait in Parkinson's disease: the impact of dual-tasking and turning. Mov Disord. (2010) 25:2563–70. 10.1002/mds.23327 20632376

[23] Beaulne-Séguin Z Nantel J. Conflicting and non-conflicting visual cues lead to error in gait initiation and gait inhibition in individuals with freezing of gait. Gait Posture. (2016) 49:443–7. 10.1016/j.gaitpost.2016.08.002 27525821

[24] Vandenbossche J Deroost N Soetens E Spildooren J Vercruysse S Nieuwboer A. Freezing of gait in Parkinson disease is associated with impaired conflict resolution. Neurorehabil Neural Repair. (2011) 25:765–73. 10.1177/1545968311403493 21478498

[25] Fasano A Schlenstedt C Herzog J Plotnik M Rose FEM Volkmann J. Split-belt locomotion in Parkinson's disease links asymmetry, dyscoordination and sequence effect. Gait Posture. (2016) 48:6–12. 10.1016/j.gaitpost.2016.04.020 27477701

[26] Ma J Gao L Mi T Sun J Chan P Wu T. Repetitive transcranial magnetic stimulation does not improve the sequence effect in freezing of gait. Parkinsons Dis. (2019) 2019:1–8. 10.1155/2019/2196195 31275542 PMC6589230

[27] Tinaz S Pillai AS Hallett M. Sequence effect in Parkinson's disease is related to motor energetic cost. Front Neurol. (2016) 7:24. 10.3389/fneur.2016.00083 27252678 PMC4877367

[28] Kang SY Sohn YH. Effectiveness of exercise on the sequence effect in parkinson's disease. J Mov Disord. (2020) 13:213–17. 10.14802/jmd.20045 32854485 PMC7502303

[29] Postuma RB Berg D Stern M Poewe W Olanow CW Oertel W. MDS clinical diagnostic criteria for Parkinson's disease. Mov Disord. (2015) 30:1591–601. 10.1002/mds.26424 26474316

[30] Goetz CG Tilley BC Shaftman SR Stebbins GT Fahn S Martinez-Martin P. Movement disorder society-sponsored revision of the unified parkinson's disease rating scale (MDS-UPDRS): scale presentation and clinimetric testing results. Mov Disord. (2008) 23:2129–70. 10.1002/mds.22340 19025984

[31] Turner-Stokes L Nyein K Turner-Stokes T Gatehouse C. The UK FIM+FAM: development and evaluation. Clin Rehabil. (1999) 13:277–87. 10.1191/026921599676896799 10460115

[32] Tofani M Massai P Fabbrini G Berardi A Pelosin E Conte A. Psychometric properties of the italian version of the barthel index in patients with parkinson's disease: a reliability and validity study. Funct Neurol. (2019) 34:145–150. Available online at: https://pubmed.ncbi.nlm.nih.gov/32453995/ (accessed May 13, 2021). 32453995

[33] De Ridder R Lebleu J Willems T De Blaiser C Detrembleur C Roosen P. Concurrent validity of a commercial wireless trunk triaxial accelerometer system for gait analysis. J Sport Rehabil. (2019) 28:295. 10.1123/jsr.2018-0295 30747572

[34] Mak MK Wong-Yu IS Shen X Chung CL. Long-term effects of exercise and physical therapy in people with Parkinson disease. Nat Rev Neurol. (2017) 13:689–703. 10.1038/nrneurol.2017.128 29027544

[35] Berra E De Icco R Avenali M Dagna C Cristina S Pacchetti C. Body weight support combined with treadmill in the rehabilitation of parkinsonian gait: a review of literature and new data from a controlled study. Front Neurol. (2019) 9:1066. 10.3389/fneur.2018.01066 30800095 PMC6375880

[36] Keus S Munneke M Graziano M Paltamaa J Pelosin E Domingos J. European physiotherapy guideline for Parkinson's disease. Kngf. (2014) 1:1–91. 10.3233/JPD-181383 32761506

[37] Vaughan-Graham J Cott C Wright FV. The Bobath (NDT) concept in adult neurological rehabilitation: what is the state of the knowledge? A scoping review. Part II: Intervention studies perspectives. Disabil Rehabil. (2015) 37:1909–28. 10.1016/j.physio.2015.03.1588 25427891

[38] Alexandre de Assis IS Luvizutto GJ Bruno ACM Sande de Souza LAP. The proprioceptive neuromuscular facilitation concept in parkinson disease: a systematic review and meta-analysis. J Chiropr Med. (2020) 19:181–7. 10.1016/j.jcm.2020.07.003 33362441 PMC7750824

[39] Castiglia SF Tatarelli A Trabassi D De Icco R Grillo V Ranavolo A. Ability of a set of trunk inertial indexes of gait to identify gait instability and recurrent fallers in Parkinson's disease. Sensors. (2021) 21:3449. 10.3390/s21103449 34063468 PMC8156709

[40] Paul SS Allen NE Sherrington C Heller G Fung VSC Close JCT. Risk factors for frequent falls in people with Parkinson's disease. J Parkinsons Dis. (2014) 4:699–703. 10.3233/JPD-140438 25271238

[41] Plotnik M Hausdorff JM. The role of gait rhythmicity and bilateral coordination of stepping in the pathophysiology of freezing of gait in Parkinson's disease. Mov Disord. (2008) 23:S444–50. 10.1002/mds.21984 18668626

[42] Espay AJ Giuffrida JP Chen R Payne M Mazzella F Dunn E. Differential response of speed, amplitude, and rhythm to dopaminergic medications in Parkinson's disease. Mov Disord. (2011) 26:2504–8. 10.1002/mds.23893 21953789 PMC3318914

[43] Desmurget M Grafton ST Vindras P Gréa H Turner RS. The basal ganglia network mediates the planning of movement amplitude. Eur J Neurosci. (2004) 19:2871–80. 10.1111/j.0953-816X.2004.03395.x 15147320

[44] Nachev P Kennard C Husain M. Functional role of the supplementary and pre-supplementary motor areas. Nat Rev Neurosci. (2008) 9:856–69. 10.1038/nrn2478 18843271

[45] Agostino R Berardelli A Formica A Stocchi F Accornero N Manfredi M. Analysis of repetitive and nonrepetitive sequential arm movements in patients with Parkinson's disease. Mov Disord. (1994) 9:311–4. 10.1002/mds.870090305 8041371

[46] Jenkins IH Jahanshahi M Jueptner M Passingham RE Brooks DJ. Self-initiated versus externally triggered movements. II. The effect of movement predictability on regional cerebral blood flow. Brain. (2000) 123:1216–28. 10.1093/brain/123.6.1216 10825359

[47] Nieuwboer A Rochester L Müncks L Swinnen SP. Motor learning in Parkinson's disease: limitations and potential for rehabilitation. Park Relat Disord. (2009) 15:53–8. 10.1016/S1353-8020(09)70781-3 20083008

[48] Johansson H Hagströmer M Grooten WJA Franzén E. Exercise-induced neuroplasticity in parkinson's disease: a metasynthesis of the literature. Neural Plast. (2020) 2020:8961493. 10.1155/2020/8961493 32256559 PMC7079218

[49] Serrao M Chini G Caramanico G Bartolo M Castiglia SF Ranavolo A. Prediction of responsiveness of gait variables to rehabilitation training in Parkinson's disease. Front Neurol. (2019) 10:826. 10.3389/fneur.2019.00826 31428039 PMC6688512

